# A Whale Tale: Using Blubber Biopsies to Characterize Pacific Ocean Pollutant Trends

**DOI:** 10.1289/ehp.119-a133b

**Published:** 2011-03

**Authors:** Angela Spivey

**Affiliations:** **Angela Spivey** writes from North Carolina about science, medicine, and higher education. She has written for *EHP* since 2001 and is a member of the National Association of Science Writers

Expression of the enzyme CYP1A1 in the skin of marine mammals has been shown by multiple studies to indicate exposure to organic pollutants in a dose-dependent manner. A new large-scale monitoring study investigated whether analysis of dermal CYP1A1 expression and organic pollutants in sperm whales (*Physeter macrocephalus*) could reveal oceanwide geographical trends in chemical exposure **[*EHP*** **119(3):337–343; Godard-Codding et al].** This is the first known study to assess broad geographic trends in CYP1A1 expression, stable carbon and nitrogen isotopes, and organic pollutant burdens in a threatened whale species.

The authors used immunochemistry to analyze CYP1A1 expression in skin and blubber samples collected from 234 sperm whales from five Pacific Ocean regions. Variation in the whales’ trophic level (position in the food chain) was examined by using mass spectrometry to measure nitrogen isotopes in skin samples; enrichment of an animal’s tissue nitrogen is known to occur as the animal eats higher on the food chain. The general latitude frequented by the whales—a reflection of where the whales were likely to have been exposed to pollution—was determined by analyzing carbon isotope ratios.

The whales exhibited significant regional differences in CYP1A1 expression. Expression was highest among whales from the Galapagos Islands, a United Nations World Heritage marine reserve, and lowest among whales from sites farthest away from continents. Differences in the whales’ age, sex, and diet did not appear to explain regional differences but could not be ruled out unequivocally.

This study did not show a significant correlation between CYP1A1 expression in skin cells and actual pollutant burden in blubber, as measured by analyzing eight sex-specific pooled samples for burdens of polycyclic aromatic hydrocarbons, hexachlorobenzene, polychlorinated biphenyls, and the pesticide DDT, then comparing them with CYP1A1 immunohistochemistry scores estimated for the pooled samples.

However, the small size of the individual biopsies allowed under standards for humane biopsying of marine mammals prevented detailed chemical analyses and limited the power to detect significant associations. Also, the biopsies were limited to the outer blubber layer, which is less metabolically active than deeper tissue. Studies in bottlenose dolphins have shown that CYP1A1 expression in the skin is more strongly related to pollutants measured in deeper blubber than in blubber closer to the skin surface; whether such stratification happens in other cetaceans requires further study.

The study succeeded at identifying regional differences in CYP1A1 expression, providing a baseline for this known biomarker of exposure to organic pollutants. Future studies that profile CYP1A1 expression in cetacean skin biopsies oceanwide are warranted to explore the global distribution of biochemically relevant levels of these chemicals.

## Figures and Tables

**Figure:**
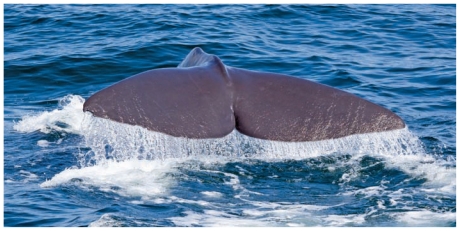
Carnivorous sperm whales are at the top of their food chain.

